# Supplementary material for: T Cell-Induced Colitis Is Exacerbated by Prolonged Stress: A Comparison in Male and Female Mice
Source: Biomedicines. 2024 Jan 18;12(1):214. doi: 10.3390/biomedicines12010214 (PMC10813177; doi:10.3390/biomedicines12010214)
Supplement: Supplementary file 1 [file biomedicines-12-00214-s001.zip › biomedicines-2773994-supplementary.pdf]

Supplemental Table S1. Primer sequences

Genes	Sequence (5'-3')	Annealing Temperature (°C)
**TNF-α**	F: AGGCACTCCCCCAAAAGATG<br>R: GTAGACAGAAGAGCGTGGTGG	60
**INF-γ**	F: GGTCAACAACCCACAGGTCC<br>R: ACTCCTTTTCCGCTTCCTGAG	60
**IL-1β**	F: GCACTACAGGCTCCGAGATGAAC<br>R: TTGTCGTTGCTTGGTTCTCCTTGT	60
**iNOS**	F: AGTGAAAAGTCGAGCCGCA<br>R: ACAATCCACAACTCGCTCCA	60
**IL-2**	F: CCTGAGCAGGATGGAGAATTACA<br>R: TCCAGAACATGCCGCAGAG	60
**IL-6**	F: GACTGATGCTGGTGACAAC<br>R: ATCCTCTGTGAAGTCTCCTC	60
**IL-7**	F: ATTATGGGTGGTAGCCG<br>R: GTTCCTGTCATTTTGTCCAATTCA	60
**IL-17A**	F: ACTACCTCAACCGTTCCA<br>R: GAGGGATATCTATCAGGGTC	60
**IL-22**	F: CCTGACCAAACTCAGCAATC<br>R: GCCTTCTGACATTCTTCTGG	60
**IL-23a**	F: CAAGGACAACAGCCAGTTCTGCTT<br>R: AGGCTCCCCTTTGAAGATGTCAGA	60
**EEF2**	F: TGTCAGTCATCGCCCATGTG<br>R: GGAGATGGCGGTGGATTTGA	60
